# Data structures and compression algorithms for high-throughput sequencing technologies

**DOI:** 10.1186/1471-2105-11-514

**Published:** 2010-10-14

**Authors:** Kenny Daily, Paul Rigor, Scott Christley, Xiaohui Xie, Pierre Baldi

**Affiliations:** 1 Department of Computer Science, University of California Irvine, Irvine, CA 92697 USA; 2 Institute for Genomics and Bioinformatics, University of California Irvine, Irvine, CA 92697 USA; 3 Department of Mathematics, University of California Irvine, Irvine, CA 92697 USA; 4 Center for Complex Biological Systems, University of California Irvine, Irvine, CA 92697 USA; 5 Department of Biological Chemistry, University of California Irvine, Irvine, CA 92697 USA

## Abstract

**Background:**

High-throughput sequencing (HTS) technologies play important roles in the life sciences by allowing the rapid parallel sequencing of very large numbers of relatively short nucleotide sequences, in applications ranging from genome sequencing and resequencing to digital microarrays and ChIP-Seq experiments. As experiments scale up, HTS technologies create new bioinformatics challenges for the storage and sharing of HTS data.

**Results:**

We develop data structures and compression algorithms for HTS data. A processing stage maps short sequences to a reference genome or a large table of sequences. Then the integers representing the short sequence absolute or relative addresses, their length, and the substitutions they may contain are compressed and stored using various entropy coding algorithms, including both old and new fixed codes (e.g Golomb, Elias Gamma, MOV) and variable codes (e.g. Huffman). The general methodology is illustrated and applied to several HTS data sets. Results show that the information contained in HTS files can be compressed by a factor of 10 or more, depending on the statistical properties of the data sets and various other choices and constraints. Our algorithms fair well against general purpose compression programs such as gzip, bzip2 and 7zip; timing results show that our algorithms are consistently faster than the best general purpose compression programs.

**Conclusions:**

It is not likely that exactly one encoding strategy will be optimal for all types of HTS data. Different experimental conditions are going to generate various data distributions whereby one encoding strategy can be more effective than another. We have implemented some of our encoding algorithms into the software package GenCompress which is available upon request from the authors. With the advent of HTS technology and increasingly new experimental protocols for using the technology, sequence databases are expected to continue rising in size. The methodology we have proposed is general, and these advanced compression techniques should allow researchers to manage and share their HTS data in a more timely fashion.

## Background

Over the past four decades, sequencing technologies have been one of the major driving forces in the life sciences producing, for instance, the full genome sequences of thousands of viruses and bacteria, and dozens of eukaryotic organisms, from yeast to man [1]. This trend is being accentuated by modern high-throughput sequencing (HTS) technologies: several human genomes were recently produced [2-5] and a project to sequence 1,000 human genomes in the next few years is under way [6]. Different HTS technologies are competing to be able to sequence an individual human genome for less than $1,000 within a few years [7] and reaching the point where human genome sequencing will be a commodity. Furthermore, not only are HTS technologies useful for sequencing and resequencing genomes, but they are also instrumental to accurately identify and measure mRNA and other nucleotide sequences in new important high-throughput applications such as digital expression microarrays, ChIP-Seq [8] and SNP genotyping. In all cases, the amount of data produced by HTS technologies as experiments scale up creates significant bioinformatics challenges to understand, store and share data. To address some of these challenges, we develop data structures and compression algorithms for the efficient management and storage of HTS data.

Several different HTS technologies have been conceived and developed to differing degrees of maturity. They can be classified into four broad classes: amplification followed by mass spectrometry, *in vitro *cloning, *in vivo *cloning, and single molecule [9]. There are currently three commercially advanced HTS systems: SOLiD (Applied BioSystems), Solexa (Illumina), 454 (Roche), all based on the *in vitro *cloning approach. Each system depends on a sheared DNA sample which is diluted onto some type of matrix, clonally amplified, and then transformed via repetitive enzymatic cycles into a series of four distinct fluorescent signals (each representing a different base) monitored at each cycle by a CCD camera. The series of fluorescent signals at each position is converted into a DNA sequence and a quality score for each position. A typical run (or lane) can generate tens of millions of sequence reads, and with a set of experiments that includes biological replicates, control and treatment samples, etc., then the total number of reads can reach into the billions. Important variations between technologies exist, for instance in terms of the length and quality of the sequences. However, all existing systems rely on the parallel sequencing of many short sequences and produce outputs of very long lists of relatively short sequences. Thus the fundamental problem we wish to address is the storage and compression of such lists.

Because of the variations that may exist between different technologies and different constraints associated with different deployment scenarios, our goal is not to provide a single solution, but to describe general methods by which customized solutions can be developed. Thus after presenting the basic idea, we review several relevant representations and compression algorithms. The approach is illustrated on several HTS data sets.

## Methods

### General Approach

In the standard text format, a file of *N *short sequences of average length *l *requires *N*(*l *+ 1) bytes (or 8*N*(*l *+ 1) bits) to store, using one ASCII byte per character, and including a character (carriage return) to separate two consecutive sequences. The ranges of *N *and *l *can vary depending on the experiment and the technology, but to fix the ideas one can imagine current values of *N *in the 10^5 ^- 10^9 ^range and of *l *in the 10^1 ^- 10^3 ^range, with most typical values in the 20-100 range. Additional information regarding, for instance, the quality of the sequences can be included in the output files.

To store and compress this information, we imagine first that the short sequences can be mapped to a reference genome. This is the typical situation for resequencing experiments, including large-scale sequencing of diploid human genomes using mapping software such as Illumina's ELAND, MAQ [10], ZOOM [11], or Bowtie [12]. In this case, each short sequence *s_i _*can be represented by its address *a_i _*in the reference genome. If the length of each sequence is not fixed and known in advance or stored in the header of the file, the length of the sequence *l_i _*must also be included. If the match is not exact, variations from the genomic sequence must also be included by recording their address and type. For simplicity, we will assume only substitutions. Thus something like "(1500, 25, 3C)" could be used to record a short sequence whose starting point matches position 1500 in the reference genome, with a length of 25 nucleotides, and a substitution by a C in position 3. Relative addresses, rather than absolute addresses, can be used not only to record variations within sequences, but also for the address of the sequences themselves. The same sequence could be encoded by "(100,25,3C)" to indicate that it is found 100 nucleotide downstream of the previously occurring sequence, provided the file has been preprocessed to reorder the sequences linearly along the genome. With relative addresses, the dynamic range of the integers to be encoded may be considerably smaller than with absolute addresses. If a sequence can be mapped equally well to multiple locations on the genome, any one of them can be chosen to represent the sequence. Finally, specific experiments could come with additional information. For instance, in SNP mapping experiments, the locations and types of variations could be constrained and leveraged to increase compression. It is worth noting that for this approach, the availability of a reference genome is not as restrictive as it may seem; minimally, a reference of DNA sequences to which experimental sequences can be reasonably mapped is needed. For simplicity in this work, we focus on the case of interest where a reference genome is available.

The idea of compressing DNA sequences is not new. Compression algorithms such as Biocompress-2 [13], CTW [14], OffLine [15], DNACompress [16] and DNA compressor [17] consider the task of directly compressing large sequence strings. While others consider alternative compression tasks such as COIL [18] which compresses a large database of unrelated sequences or DNAzip [19] which compresses variations to a reference genome. Our goal is different as we want to compress a large number of very short sequences while using a large reference sequence. Whether or not it is advantageous over the standard text format, however, depends on the details of the implementation and, more often than not, the data being compressed. A successful implementation of the basic idea depends crucially on careful consideration of the encoding scheme. In particular, the choice of the function converting integers to binary strings, has a great effect on the resulting compression. For our application, these integers are the absolute or relative addresses and lengths of sequence reads. It is essential to understand that simply converting integers to their binary value (e.g. converting "25" to "11001") does not work since one does not know where one integer ends and the next starts. No symbol other than 0 or 1 is available to separate consecutive integers. Furthermore, such a simple encoding scheme does not take into account any entropy considerations. Likewise, a general purpose compression scheme for text data, such as Lempel-Ziv (gzip [20]), is likely to be far from optimal for HTS data. Thus we are interested in binary encoding schemes for sequences of integers that can be parsed automatically and, consistent with information theory, are entropy efficient, in the sense that fewer bits are used to encode more frequent events.

A simple back-of-the-envelope calculation, however, can show why the situation is hopeful. Suppose the information associated with the integer *j *representing an address can be stored in about 2 log *j *bits (all logarithms are taken to the base 2). This corresponds to a penalty factor of two over the plain binary encoding and can be achieved with the coding methods described in the next section. Then the equality 8(*l *+ 1) = 2 log *j *shows that some degree of compression is achieved as long *j *is less than 2^4(*l*+1)^. Even with *l *as small as 20, this yields 2^84 ^which is much larger than the length of any genome. Assuming that the length of each sequence must also be stored, this may require at most a fixed number of bits *C*. If the lengths are between 20 and 36, for instance, they can be described with 4 bits. From the relation 8(*l *+ 1) = 4 + 2 log *j *we find again that compression is achievable as long as *j *is less than 2^4*l*+2^, which is again easily achieved in the current environment. Furthermore, with for instance *l *= 24 these relations show that a 20-fold or so compression rate should be achievable with reasonable values of *j*. A similar calculation can be made including information about the number of substitutions, their locations, and types for each sequence.

### Specific Encoding Strategies

To begin with, we illustrate these issues here by considering how the integer addresses *a_i_*, relative or absolute, can be encoded into a binary string. From Shannon's entropy coding theory [21,22], optimal encoding of these integers from a compression standpoint depends on their distribution in order to assign shorter binary codes to more probable symbols (integers). For simplicity, we distinguish two broad classes of codes: fixed codes, such as Golomb [23] and Elias codes [24] and their more recent variants [25], and variable codes, such as Huffman codes [26]. In a fixed code, the integer *i *is always encoded in the same way, whereas in a variable code the encoding changes.

#### Fixed Codes: Golomb and Golomb-Rice Codes

Both Golomb codes and Elias codes encode an integer *j *by catenating two bit strings: a preamble *p*(*j*), that encodes *j*'s scale, and a mantissa. Golomb codes were specifically developed to encode stationary coin flips with *p *≠ 0.5. Thus they are known to be optimal and asymptotically approach the Shannon limit if the data is generated by random coin flips or, equivalently, if the distribution over the integers is geometric, although they can be used for any other distribution. The more skewed the probability *p *is (towards 0 or 1) the greater the level of compression that can be achieved.

Golomb codes have one integer parameter *m*. Given *m*, any positive integer *j *can be written using its quotient and remainder modulo *m *as *j *= ⌊*j/m*⌋ + (*j *mod *m*). To encode *j*, the Golomb code with parameter *m *encodes the quotient and remainder by using:

• ⌊*j*/*m*⌋ 1-bits for the quotient;

• followed by a 0, as a delimiter (unary encoding of ⌊*j*/*m*⌋);

• followed by the phased-in binary code for *j *mod *m *for the remainder (described below).

The encoding of integers 0, ..., *m *- 1 normally requires *B *= ⌈log *m*⌉ bits. If *m *is not a power of two, then one can sometimes use *B *- 1 bits. More specifically, in the "phased-in" approach:

• if *i <*2*^B ^*- *m*, then encode *i *in binary, using (*B *- 1) bits;

• if *i *≥ 2*^B ^*- *m*, then encode *i *by *i *+ 2*^B ^*- *m *in binary, using *B *bits.

For instance, for *m *= 5, *i *= 2 is encoded as "10" using 2 (= *B *- 1) bits, and *i *= 4 is encode as "111" using 3 (= *B*) bits. Thus the encoding of *j *requires in total ⌊*j/m*⌋ + 1 + ⌊log *m*⌋ or ⌊*j/m*⌋ + 1 + ⌈log *m*⌉ bits and the codeword for the integer *j *+ *m *has one more bit than the codeword for the integer *j*. Unless otherwise specified, all logarithms are taken to base 2. We use also "[log *m*]" to denote "⌊log *m*⌋.or ⌈log *m*⌉".

Finally, Golomb-Rice codes are a particularly convenient sub-family of Golomb codes, when *m *= 2*^k^*. To encode *j*, we concatenate ⌊*j/*2*^k^*⌋ 1-bits, one 0-bit, and the *k *least significant bits of *j*. The length of the encoding of *j *is thus ⌈*j/*2*^k^*⌉ + *k *+ 1. The decoding of Golomb-Rice codes is particularly simple. First, read and count the number of 1 bits until the first 0 bit is found. The number of 1 bits is the quotient *q *= ⌊*j/m*⌋. Then, read the next log *m *bits to get the binary representation of the remainder *r *= *j *mod *m*. The decoded value equals *j *= (q * m) + *r*.

#### Elias Codes

In the Elias Gamma coding scheme, the preamble *p*(*m*) is a string of zeroes of length ⌊log *j*⌋, and the mantissa *m*(*j*) is the binary encoding of *j*. More precisely, to encode the scale and value of *j*:

• write ⌊log *j*⌋ 0-bits;

• followed by the binary value of *j *beginning with its most significant 1-bit.

The length of the encoding of *j *is 2⌊log *j*⌋ + 1(Table 1). The decoding is obvious: first read *n *0-bits until the first 1-bit is encountered, then read *n *more bits to get the binary representation of *j*.

**Table 1:** Example of Elias Gamma (EG) Coding.

Number	Encoding
1	1
2-3	01x
4-7	001xx
8-15	0001xxx
16-31	00001xxxx

Each integer *j *is encoded by concatenating ⌊log *j*⌋ 0's with the binary value of *j*

Applying the relationship

(1) −logP(j)≈2⌊logj⌋+1

to the integer probabilities, shows that Elias Gamma encoding asymptotically approaches the Shannon limit for *P *(*j*) ≈ *Cj*^-2^. This is a power law relationship with exponent -2 and *C *is a normalizing constant. Note that for both Golomb and Elias Gamma codes, several different consecutive integers can be encoded into a bit vector with the same length, hence the relationships -log *P *(*j*) ≈ length(*j*) is only approximate with respect to geometric or power-law distributions over the integers.

#### Monotone Value Coding (MOV Coding)

More recently, new families of efficient fixed codes for integers have been developed [25,27-29], for instance in the case of increasing or quasi increasing sequences of integers, by encoding only the deltas of the preambles. Here we introduce a modification of the codes described above, presented with the Elias Gamma codes, for messages consisting of monotone sequences of integers, such as consecutive absolute addresses of sequence reads. When the value of the integers being encoded increases monotonically, additional lossless compression can be obtained by encoding only the scale increases and their location (Table 2).

**Table 2:** Example of Monotone Value (MOV) Coding.

Number	Encoding
1	1
2	10
3	11
9	1001
14	1110
26	11010
29	11101

The principle is illustrated using the vector of addresses (1, 2, 3, 9, 14, 26, 29). Each integer *j *is converted to a binary representation of length ⌊log *j*⌋ which begins with a 1-bit. 0-bits are used between two consecutive integers only when the length (scale) increases. The number of 0-bits is equal to the increase in the length. The final encoding of the vector is 1 0 10 11 00 1001 1110 0 11010 11101

More precisely, if a sequence of increasing addresses is given by (*j*_1, _*j*_2, _..., *j_K_*) with *j*_1 _*< j*_2 _... <*j_K_*:

• encode *j*_1 _using Elias Gamma encoding;

• for *i *= 2, ..., *K*:

- write ⌊log *j*_*i*_⌋ - ⌊log *j*_*i*-1_⌋ 0-bits;.

- followed by the binary value of *j_i _*beginning with its most significant 1-bit.

The MOV-encoded vector of addresses can be decoded by a simple algorithm:

• set *k *= 1;

• decode each integer in succession by repeating the following steps:

• increment *k *by the number of 0-bits in the input stream before reaching the first 1-bit;

• counting this first 1-bit as the first digit of the integer, read the remaining *k *- 1 bits of the integer from the input stream.

Another variation called Monotone Length Coding (MOL Coding) can be used for quasi-monotone sequence tolerating occasional deviations from a monotone behavior [25]. Another scheme that may be useful for encoding integers but cannot be described for conciseness reasons is the Binary Interpolation [27] scheme, together with several variants.

#### Variable Codes

In genomic applications, in general the integers may not have a well defined distribution, in which case it is always possible to use a general entropy encoding scheme, such as Huffman coding [21,22,26] which essentially builds a prefix code by using a binary hierarchical clustering algorithm starting from the events (integers) with the lowest probability. While Huffman coding achieves compression close to the entropy limit, the price to pay over fixed coding schemes such as Golomb and Elias Gamma, or the more recent codes mentioned above, is the storage of the Huffman table which can be quite large in some applications. However this is a fixed cost with respect to the database size, and therefore whether this cost is acceptable or not depends on the specific application. Small gains in compression over Huffman coding may be obtained using arithmetic coding [30,31], but at a non-trivial price in the complexity of computations. For more information about integer encodings, refer to references [32] and [33].

#### Byte Arithmetic

Direct implementations of the decoding algorithms process the compressed representations bit-by-bit; however, it is possible to implement faster decoders, which decode the compressed data byte-by-byte. These faster decoders work by looking up information from pre-computed tables. These tables are indexed by: (1) all possible bytes *B *(ranging from 0 to 255); and (2) a bit-index *i *(ranging from 0 to 7) which marks the position of the decoder within the byte. These tables may store quantities such as the binary value of byte *B *starting from bit *i*, the number of bits turned on in byte *B *starting from bit *i*, and the unary value of byte *B *starting from bit *i*. The exact quantities stored depend on the details of a particular decoder implementation.

In practice, byte arithmetic considerably increases decoding speed, sometimes approaching as much as an eight-fold improvement over the corresponding bit-by-bit implementation. The exact value of the speedup depends on several factors including the characteristic of the data, the exact compression scheme, and the hardware used.

## Results and Discussion

For conciseness, we present a subset of representative results focusing on Elias, MOV and Variable codes. Golomb code results are in general comparable to Elias Gamma results, typically with a slight decrease in performance. Furthermore, Golomb's code require tuning one additional parameter (*m*) and thus are not reported here.

For each data set, we transformed the data into a uniform flat file format, separating the location information from the mismatch information for each read, then performed encoding on the location and mismatch information separately. The following sections describe the different encoding strategies used for location and mismatch data and their corresponding results.

### Data Extraction and Statistics

We selected three data sets representative of typical short read sequence data derived from different experimental settings aimed at addressing different biological questions, from genome sequencing to transcription factor binding site mapping. Each of the data sets correspond to a different combination of genome coverage, repetitiveness and locational specificity in the genome, so that our encoding results provide insights into how different strategies can be applied and tailored to different data. Table 3 gives some basic statistics for each data set, including the sizes for the original standard text format for the sequence reads, the uniform flat file format as described above, and the Bowtie alignment output. The uniform flat file format sizes are further split into the sizes of the location data and mismatch data. The Bowtie alignment output contains additional information beyond the minimal location and mismatch data required to reconstitute the reads, so Table 3 provides compression sizes of that additional information with a set generic compression tools. Those sizes will be used later for comparison of compression results for Bowtie output.

**Table 3:** Statistics of Three High-Throughput Data Sets

	Dataset 1	Dataset 2	Dataset 3
Reads (× 10^6^)	6.4	1.7	31
Read length	19	25	23-44
Coverage	Very sparse	Sparse	Full

File sizes			
Raw Sequence	1,030,333,440	353,181,920	8,869,613,392
Uniform	912,352,288	252,540,968	4,946,059,912
Location	743,517,128	226,557,032	4,232,120,216
Mismatches	168,835,160	25,983,936	713,939,696
Bowtie	3,145,664,248	902,954,872	19,475,952,512

Bowtie Extra Fields			
gzip	50,382,904	106,576,328	839,247,848
7zip	36,306,064	93,238,688	778,347,264

#### Dataset 1

The first data set is obtained from the laboratory of Dr. Suzanne Sandmeyer at University of California Irvine and comes from an experiment aimed at mapping retrotransposon Ty3 insertion sites in the yeast genome. It consists of 6,439,584 sequence reads, all of length 19 bp. By the nature of the underlying experiments, the sequences in this data set are highly clustered, often with a high degree of repetition. The reads have at most two substitutions. The numbers of sequences with 0, 1, and 2 substitutions are given by 3,468,077 (54%), 895,997 (14%), and 2,075,510 (32%) respectively.

#### Dataset 2

**Figure 1:**
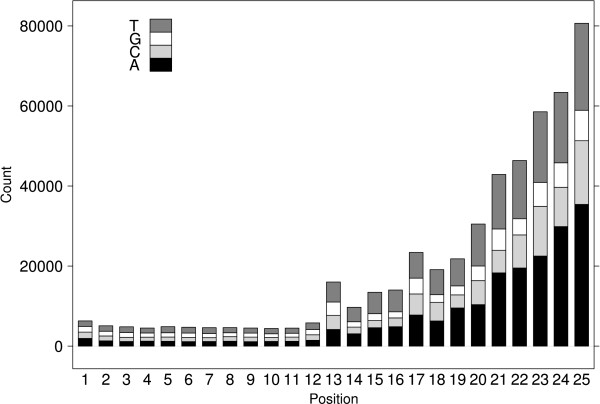
**Dataset 2 Nucleotide Substitutions**. Distribution of nucleotide substitutions at each read position in Dataset 2. The shading of the bar indicates which nucleotide was present in the read.

The second data set comes from a chromatin immunoprecipitation assay (ChIP-Seq) used to map the *in vivo *binding site locations of the neuron-restrictive silencer factor (NRSF) in humans [34]. It consists of 1,697,990 sequence reads, all of length 25 bp and mapped to the most recent human genome sequence (hg18). The reads have at most two substitutions. The numbers of sequences with 0, 1, and 2 substitutions are given by 1,297,153 (76%), 302,939 (18%), and 97,899 (6%) respectively. Figure 1 shows the number of mismatches found at each position along the read, as well as the types of substitutions. The number of mismatches increases towards the end of the read, as expected with the Solexa sequencing technology where the error rate increases further along the length of the read. Our interest is encoding the read sequence as is without attempting to differentiate between true SNPs and sequencing errors, but the distribution clearly shows that the majority of these mismatches are observed at the end of the read which can be used to advantage when encoding the variations.

#### Dataset 3

The third data set corresponds to a full diploid human genome sequencing experiment for an Asian individual [5]. This is a very large data set with enough reads to provide 36-fold average coverage, and we utilized the existing mapping of the reads provided by the YH database [35] to the human reference genome. For illustrative purposes, we report only the results corresponding to the reads associated with chromosome 22. For chromosome 22, there are 31,118,531 reads that vary in length from 30 to 40 bp for a total of 1,108,701,700 bp of sequence data. The numbers of sequences with 0, 1, and 2 substitutions are given by 19,126,772 (61%), 6,166,549 (20%), and 5,825,210 (19%) respectively.

### Encoding of Location Information

The location information for a mapped read consists of a chromosome identifier, a position along that chromosome, the strand, the length of the read, and the number of mismatches it contains. In the flat file format, each read is specified on a single line with the values separated by a comma. One technique is to encode each of the attributes individually. For this standalone technique, we compute the frequency of occurrence of each of the attributes, order them, and then use EG encoding on their ordered index. An alternative method is to combine all of the attributes together. The (*C*, *S*, *M*)*Lookup *method takes the attributes combined together as tuples, for example (chromosome, strand, number of mismatch), then computes the frequency of a subset of these. The combination method of REG Indexed is described in detail below.

Table 4 gives the comprehensive set of compression algorithm results. For the data sets where all of the reads have the same length (1 and 2), we omit the length of the read and assume it is specified in a header structure for the data. The top part of Table 4 shows the standalone and combined techniques for encoding the location information, while the bottom part of Table 4 is for encoding the mismatch information described in the next section. For the standalone methods, multiple encoding techniques are tried for the start location while only EG encoding is used on the other attributes. The best standalone compression attainable is also shown; this should be compared to the best compression attainable by the either of the combined methods to determine which encoding method to use for encoding the start location. This in combination with the mismatch encoding gives the best total compression for the dataset. Throughout the table, the best compression for a specific technique is shown in italics.

**Table 4:** Compression Algorithm Results on Three High-Throughput Data Sets

	Dataset 1	Dataset 2	Dataset 3
Standalone Methods			
Read Length	6,439,584	1,697,990	59,267,219
Chromosome	31,576,860	9,997,062	31,118,531
Strand	6,439,584	1,697,990	31,118,531
# Mismatches	12,382,598	2,499,664	55,624,291
**Total**	**50,399,042**	**14,194,716**	**117,861,353**
Start Location			
MOV^†^	121,565,953	44,200,254	787,554,494
EG^†^	236,691,716	86,701,276	1,543,990,407
REG^†^	10,745,562	26,180,752	76,430,489
Huffman	91,019,189	82,444,521	1,324,964,740
RHuffman	*10,311,095*	*19,066,500*	*65,905,674*

**Best Standalone**	**60,710,137**	**33,261,216**	**183,767,027**

Combined Methods			
(C,S,M) Lookup	64,424,309	33,809,380	158,272,463
REG Indexed^†^	*12,133,110*	*32,342,080*	*144,975,985*

Mismatches			
Nucleotide	13,917,023	1,307,870	53,441,350
From Start	30,028,807	4,177,576	159,433,004
From End	32,671,455	2,333,372	153,865,294
Total Start	*43,945,830*	5,485,446	212,874,354
Total End	46,588,478	*3,641,242*	207,306,644
Combined^†^	44,033,309	3,757,400	*186,298,126*

**Best Compression**	**56,078,940**	**35,983,322**	**390,541,330**
**GenCompress**	**56,166,419**	**36,099,480**	**390,541,330**

All of the results are based upon theoretical calculations without doing the actual encoding. In the Implementation section, we describe our GenCompress software package which implements some of the encoding methods. The methods implemented in GenCompress are marked with a † in Table 4, and the best compression achieved by GenCompress is also shown. In the following, we describe in more detail the various methods used for encoding the location information.

1. Elias Gamma (EG) Absolute: We assume that the reads cannot be reordered in any way and thus must be processed exactly as specified. The chromosome, absolute start coordinate, strand integer values, and for Dataset 3 the read length were encoded using Elias Gamma codes.

2. Elias Gamma (REG) Relative: We assume that the reads can be ordered in any way in order to achieve better compression results. We group all of the reads for each chromosome together. Within each chromosome, the reads are sorted by increasing position number; therefore the relative distance between adjacent reads is encoded rather than their absolute positions. These relative addresses correspond in general to significantly smaller integer values than the absolute addresses, especially for long chromosomes or reads with high-coverage. The chromosome, strand integer values, number of mismatches, and the read length (only for Dataset 3), were encoded using Elias Gamma codes.

3. Relative Elias Gamma Indexed (REG Indexed): We again assume that the reads can be ordered in any way in order to achieve better compression results. We group all of the reads for each chromosome together, then group reads for each strand together within a chromosome, and further group them for the number of mismatches they contain. Within each bin of (chromosome, strand, number of mismatches), we then encode the relative distances as stated above. Because the reads are grouped by chromosome, strand, and number of mismatches, there is no need to encode that information for each read. Instead, those values are stored along with a count of the number of reads for that group in a header structure. Thus, except for the additional read length information for Dataset 3, only the relative distances between the reads are encoded using Elias Gamma.

4. Monotone Value (MOV): Like the EG Relative encoding above, we reorder the reads for Monotone Value encoding according to chromosome and position. However, we use MOV codes for the absolute locations as the positions are now in increasing order.

5. Huffman: We can use the start positions (or relative starts, denoted RHuffman) to compute a Huffman tree which we use to encode. The resulting size encoding with this method also includes storing the Huffman tree, which is needed for decoding.

Table 4 shows that significant compression of the location information is achieved for all three data sets. The REG Indexed encoding was best for all data sets. Slightly better compression can be obtained on Dataset 1 by only using unique positions in the chromosome, and encoding the count of the number of occurences, (unique relative Elias Gamma encoding), which might be expected from the highly clustered and repetitive nature of the data (data not shown). While the Relative Huffman coding (RHuffman) performs the best compression on the actual start position integers, the other columns of the location must also be stored, making this method worse than the REG Indexed method. On Dataset 1, the size of the alphabet being encoded (the possible relative start distances) is small, due to the extreme sparseness of the data. Huffman coding is known to be less efficient on this type of data. As the size of the alphabet increases, Huffman coding performs better relative to the run length encoding methods (Dataset 2 has the largest alphabet to use for building the Huffman tree, and has the largest gain in performance relative to the other methods). The high compression of almost 25 fold of data for the human genome sequencing project (Dataset 3) is very encouraging as these are some of the largest data sets being generated. This data set corresponds to much of the data being generated by the 1000 genomes project. The ChIP-Seq data (Dataset 2) has the lowest compression fold of the three data sets, yet even so Table 4 shows that the total encoding of the data achieves over an order of magnitude reduction in size, significantly better than gzip.

### Encoding of Mismatch Information

The mismatch information for a mapped read consists of the positions of one or two mismatches located somewhere along the read and the nucleotide value (A, C, G, T) for the mismatch. The flat file format for mismatch information has one read per line; the line is blank if no mismatches otherwise it contains a comma separated list of mismatches (e.g. 19A,22C for two mismatches).

The sequence mismatches can be encoded in multiple ways. One possibility is to encode the position of the mismatch directly from the start of the read. However, the mismatches have a clear tendency to occur towards the end of the read as illustrated in Figure 1, so measuring the mismatch from the end of the read can reduce the number of bits to encode the position (i.e. a mismatch at position 24 of a read of length 25 would be measured as 1). For both strategies, the position is encoded using Elias Gamma codes.

The nucleotide substituted at a particular position must also be encoded; the straightforward naïve approach is to map them to integers (A to 1, C to 2, etc.). This can be optimized by ordering the nucleotides by their frequency of occurrence, so the most frequent substitution maps to the lowest integer. In both cases, the value is encoded using Elias Gamma codes.

We also tried another strategy where the position and nucleotide substitution are combined together into a single value. The combination are ordered by frequency and then encoded using Elias Gamma codes. This technique may possibly be effective if there is a large number of duplicate mismatches across all the reads.

We investigated all of these strategies and the results can be seen in Table 4. For each data set, a different method ended up having the best compression.

### Final Encoding

With the location and mismatch information combined together, we have a final encoding for a representative set of short read sequence data produced by HTS technologies. Using encoding techniques that consider the inherent structure of sequence data consistently perform well for all the data sets we tested. For our test data sets, it is interesting to note the size ratios for encoding the location information versus the mismatches. For data set 1, the mismatches clearly dominate the total compression size while the location information is very small due to the clustered nature of the reads on the genome. On the other hand, data set 2 has much fewer mismatches and the read locations are sparsely distributed across the genome, so the location information dominates the total compression size. Data set 3 is balanced between the two because it has full coverage of the genome. For data sets 1 and 3 which have a large number of mismatches, using dbSNP data as reference variation data may offer the opportunity for further compression.

### Implementation

We have implemented a subset of the encoding techniques describe in this article into a software package called GenCompress. The implementation is primarily based on the Relative Elias Gamma Indexed (REG Indexed) encoding for the location information and the Combined encoding for the mismatch information because they offer the all around compression. Currently, the quality scores are not yet encoded, and the decoded data recapitulates only the location and mismatch information. GenCompress performs two efficient passes on each dataset obtaining statistics on the mismatches during the first pass prior to actual encoding on the second pass. A more advanced data structure is currently being developed to avoid these two passes. However, this implementation allows for stream processing with minimal memory requirements. GenCompress is currently only compatible with output of the bowtie short-read aligner. A future implementation of a decoder will use the bowtie libraries to obtain the actual genomic sequences in the decoded output, and a framework exists for supporting multiple aligner suites.

Table 5 provides a comparison of the compression sizes and ratios for the three data sets for the best theoretical algorithm, our GenCompress implementation and the general purpose programs of gzip, bzip2 and 7zip. Data sizes for the raw sequence, uniform flat file format, and the output file from the bowtie alignment program are repeated from Table 3; and the compression ratios for each method is shown with respect to the different data formats. The bowtie alignment program produces additional data beyond just location and mismatch information, so that data is separated and compressed with 7zip. For GenCompress and the theoretical algorithm, the compression ratio of "Bowtie" is just for the location and mismatch information, while the "Bowtie+" ratio also includes the additional 7zip compressed data. For the gzip, bizp2 and 7zip, the "Bowtie" results are for the full bowtie alignment output file.

**Table 5:** Comparison of Compression Results

	Dataset 1	Dataset 2	Dataset 3
Original Data Sizes			
Raw Sequence	1,030,333,440	353,181,920	8,869,613,392
Uniform	912,352,288	252,540,968	4,946,059,912
Bowtie	3,145,664,248	902,954,872	19,475,952,512
Bowtie Extra Fields (7zip)	36,306,064	93,238,688	778,347,264

Best Compression	56,078,940	35,983,322	390,541,330
Raw Sequence	18	10	23
Uniform	16	7	13
Bowtie	56	25	49
Bowtie+	34	7	17

GenCompress	56,166,419	36,099,480	390,541,330
Raw Sequence	18	9	23
Uniform	16	7	13
Bowtie	56	25	49
Bowtie+	34	7	17

gzip			
Raw Sequence	41,378,624	95,688,992	618,818,824
	24	3	14
Uniform	42,918,256	54,762,528	603,836,784
	21	4	8
Bowtie	459,640,264	236,156,432	1,640,587,416
	7	4	12

bzip2			
Raw Sequence	42,233,336	94,030,320	955,061,616
	24	3	9
Uniform	36,400,576	54,656,000	649,419,632
	25	4	7
Bowtie	250,373,616	171,835,792	1,609,317,768
	13	5	12

7zip			
Raw Sequence	30,651,664	83,319,584	411,811,520
	33	4	21
Uniform	27,852,952	34,482,312	283,490,928
	33	7	17
Bowtie	247,481,992	183,522,960	1,254,167,144
	13	5	16

Table 5 indicates the results are somewhat mixed. For data set 1, the general purpose programs get a better compression than GenCompress for the raw sequence and the uniform flat file format. The number of mismatches dominates data set 2, so novel methods to more efficiently encode those mismatches would be useful. However, GenCompress is equivalent and up to 3× better then the general purpose programs for data sets 2 and 3. Interestingly in all cases, GenCompress does the best compression for the bowtie alignment data which is significant because that data ends up being larger than the original raw sequence reads by 2-3×.

Table 6 compares the timing results for compression and decompression of the data sets for GenCompress and the general purpose programs. GenCompress uses REG Indexed and combined mismatches encoding mismatches which corresponds to the compression results in Table 5, and the general purpose programs were invoked using default settings. We performed the timing of compression and decompression ten times on each dataset per method, and Table 6 provides the average timing for those ten runs. The hardware we use is a Dell T7550 with two quad core Intel Xeon E5450, 24 GB of RAM and four 146 GB SAS drives configured as RAID 1. The timing results show that both GenCompress and gzip are consistently faster than bzip2 and 7zip.

**Table 6:** Comparison of Compression and Decompression Timing

	Dataset 1	Dataset 2	Dataset 3
Compression (sec)			
GenCompress	20	5	111
gzip	10	13	70
bzip2	78	20	422
7zip	107	77	447

Decompression (sec)			
GenCompress	2	1	15
gzip	2	1	13
bzip2	7	4	53
7zip	4	2	21

## Conclusions

We have presented a set of data structures and compression algorithms for high-throughput sequencing data. We have transformed the nucleotide sequences into location and mismatch information through a mapping procedure to a reference genome, then applied fixed codes to encode that location and mismatch information in an efficient manner. We note that the mapping procedure does not need to be precise and find the correct position in the genome. We require only that a position is found because we use it only for the purpose of compressing and later decompressing the sequence. In fact, any arbitrary genome sequence can be used for mapping the reads, but it is likely that the genome which most closely matches the organism for the read data will provide the best performance. The methodology we have proposed is general, and we have illustrated its effectiveness on a representative set of HTS data. Results show that some of the information in the HTS data can be compressed by a factor of 10 or more. The proposed algorithms are comparable or slightly better than the best general compression algorithms such as bzip2 and 7zip, but those programs require a much greater processing time compared to our algorithms.

The term "post-genomic era" has become somewhat fashionable, it is clear that the genomic era is far from over, and may in fact be only at an early stage of development. With the advent of HTS technology and increasingly new experimental protocols for using the technology, the sequence databases are only expected to continue rising in size. While the local storage of this data is not especially burdensome with inexpensive hard drives of greater than 1 TB available, sharing and transferring the data is time-consuming because network speeds are an order of magnitude slower than disk. Take for example the Yan Huang genome [5] we used for Dataset 3; the total amount of read data generated by the project is almost 120 GB. Uncompressed, a typical university network can upload this data in 120 hours at roughly 1 GB per hour; that may actually take a couple of weeks of real work time by a researcher handling the upload process, and that doesn't even consider restarts that may occur due to network outages or that the network has to be shared with many other users. Extrapolating the compression results we obtained for chromosome 22 would reduce the total read data down to 5 GB, reasonable to upload in a single day. The situation becomes worse for downloading as numerous researchers may attempt to download the data, quickly saturating the server network bandwidth; advanced compression techniques such as we have introduced would allow more researchers to obtain the data in a timely fashion.

It is not likely that exactly one encoding strategy will be optimal for all types of HTS data. Different experimental conditions are going to generate various data distributions whereby one encoding strategy can be more effective than another. For encoding the location information, we have shown that two different strategies are effective. Furthermore in this article, we have only been able to consider Solexa data. It would be worthwhile to investigate both 454 data with its wide range of read lengths and SOLiD with its color space representation as the sequencing error distributions may be different for these technologies, thus affecting the mismatch locations and the strategy used for encoding them. It isn't necessary that a single strategy be picked; our software computes the respective compression metrics for all of the strategies, so they can be compared and the best one chosen automatically. We have focused exclusively on the nucleotide sequence data of the short reads from HTS; however, there is additional data that is also present including read identifiers and quality scores. A complete solution would require that this information also be encoded so that it can be recovered later. The quality scores are needed as they are used by assembly programs for determining the statistical significance of the final assembled sequence, and also by programs that call SNPs and other structural variations. Read identifiers are often just sequential numbers with little meaning, but they do become needed for cross referencing when mate paired reads are sequenced. However in both cases, the encoding techniques described in this paper can be applied to significantly reduce the size of this data.

The techniques we have described assume that the reads have already been mapped to a reference genome before they are encoded, and in our analysis we have only discussed encoding mapped reads. However in all sequencing experiments, there are reads which do not map to the reference genome. In many cases, these reads are contaminants such as bacteria and might not be relevant for the particular experiment, but in other cases those reads may be important if it is a de novo sequencing project or the reference genome is unfinished or has poor coverage. The simple solution is to use a generic compression program like gzip for those unmapped reads. Another possibility is to use multiple reference genomes. If those reads map successfully to other genomes, then equivalent levels of compression can be expected with just a small amount of header overhead required to reference the genome used.

It is also common for reads to map to multiple places on the reference genome. Our current implementation just takes the first best match, so additional optimization might be worthwhile to investigate. For example, if a read maps to one location which is very far from other reads, but has another mapping which is close to other reads, it would be advantageous to take the latter mapping because its relative starting position should be a smaller number. Optimizing all of the reads in this fashion is possibly a very difficult problem though because minimizing the distances between reads corresponds to doing clustering, and many optimal clustering algorithms are known to be NP-complete. However, approximate clustering which can be done with an online algorithm may offer some advantage. There is other data beside the read starting location which can be considered including the chromosome, strand and number of mismatches. The REG Indexed strategy performed consistently well, so taking alternative mappings for reads might create denser data bins which can be effectively encoded with fewer bits. Future research needs to consider whether the extra computational expense of performing these optimizations are a worthy compromise to the compression gains.

One might consider that because there is a lot of repetitive sequence within genomes, that an optimal encoding strategy like Huffman codes directly on the sequence itself would work very well. We investigated this possibility by encoding k-mers for data sets 1 and 2, the results can be seen in Table 7. The results seem to bear this out. Data set 1 which is a mapping experiment of retrotransposons would be expected to have considerable repetitive sequence, and Huffman coding does a very good job of compressing this data set. It does better than our algorithms and the general purpose compression programs. On the other hand, Huffman coding gets worse compression than our algorithms for data set 2.

**Table 7:** Sequence encoding using Huffman Trees.

Dataset	*k*	Sequence bits	Tree bits	Total bits
1	1	31,674,558	40	31,674,598
	2	28,340,409	324	28,340,733
	3	27,708,166	1,951	27,710,117
	4	22,565,417	10,471	22,575,888
	5	19,126,288	53,178	19,179,466
	6	21,056,658	256,303	21,312,961

2	1	94,680,841	52	94,680,893
	2	81,954,644	549	81,955,193
	3	81,038,827	4,303	81,043,130
	4	80,554,549	27,458	80,582,007
	5	83,570,470	148,206	83,718,676
	6	79,977,714	622,784	80,600,498

The data is preprocessed by counting the frequencies of *k*-mers, and this is used to build a Huffman tree. The tree is used to encode the data, and the number of bits needed to store the data as well as the tree are given

Similar encoding ideas have been applied by us to a related but different problem, the storage of entire genomes [19]. Results indicate that almost 1000-fold compression can be obtained for the human genome by encoding only the variations of the genome against a reference genome and a reference SNP database. These techniques might be further improved if we consider that most SNPs in the human genome are biallelic (exist in only one of two forms), are clustered together into haplotypes, and shared among many individuals. Instead of storing the variation for each read, a SNP map might be utilized which summarizes the variation across all of the reads or subsets of reads. Implementation requires careful consideration of the difference between true SNPs and sequencing errors, and whether an existing database like dbSNP is appropriate to use as reference (as it will not include mismatches due to sequencing errors) or a custom constructed database would work better.

## Authors' contributions

PB conceived the study and some of the basic compression algorithms. PB and XX coordinated and supervised the study. PB, KD, SC, and PR drafted the manuscript. KD and PR wrote the software and performed the simulations. All authors analyzed the results and read and approved the final manuscript.

## References

[B1] International Human Genome Sequencing Consortium Initial sequencing and analysis of the human genome Nature 2001 409 860 921 10.1038/35057062 11237011

[B2] Levy S Sutton G Ng PC Feuk L Halpern AL Walenz BP Axelrod N Huang J Kirkness EF Denisov G Lin Y MacDonald JR Pang AWC Shago M Stockwell TB Tsiamouri A Bafna V Bansal V Kravitz SA Busam DA Beeson KY McIntosh TC Remington KA Abril JF Gill J Borman J Rogers YH Frazier ME Scherer SW Strausberg RL Venter JC The diploid genome sequence of an individual human PLoS Biol 2007 5 10 e254 10.1371/journal.pbio.0050254 PMC1964779 17803354

[B3] Wheeler DA Srinivasan M Egholm M Shen Y Chen L McGuire A He W Chen YJ Makhijani V Roth GT Gomes X Tartaro K Niazi F Turcotte CL Irzyk GP Lupski JR Chinault C Song XZ Liu Y Yuan Y Nazareth L Qin X Muzny DM Margulies M Weinstock GM Gibbs RA Rothberg JM The complete genome of an individual by massively parallel DNA sequencing Nature 2008 452 7189 872 6 10.1038/nature06884 18421352

[B4] Bentley DR Balasubramanian S Swerdlow HP Smith GP Milton J Brown CG Hall KP Evers DJ Barnes CL Bignell HR Boutell JM Bryant J Carter RJ Cheetham RK Cox AJ Ellis DJ Flatbush MR Gormley NA Humphray SJ Irving LJ Karbelashvili MS Kirk SM Li H Liu X Maisinger KS Murray LJ Obradovic B Ost T Parkinson ML Pratt MR Rasolonjatovo IMJ Reed MT Rigatti R Rodighiero C Ross MT Sabot A Sankar SV Scally A Schroth GP Smith ME Smith VP Spiridou A Torrance PE Tzonev SS Vermaas EH Walter K Wu X Zhang L Alam MD Anastasi C Aniebo IC Bailey DMD Bancarz IR Banerjee S Barbour SG Baybayan PA Benoit VA Benson KF Bevis C Black PJ Boodhun A Brennan JS Bridgham JA Brown RC Brown AA Buermann DH Bundu AA Burrows JC Carter NP Castillo N Catenazzi MCE Chang S Cooley RN Crake NR Dada OO Diakoumakos KD Dominguez-Fernandez B Earnshaw DJ Egbujor UC Elmore DW Etchin SS Ewan MR Fedurco M Fraser LJ Fajardo KVF Furey WS George D Gietzen KJ Goddard CP Golda GS Granieri PA Green DE Gustafson DL Hansen NF Harnish K Haudenschild CD Heyer NI Hims MM Ho JT Horgan AM Hoschler K Hurwitz S Ivanov DV Johnson MQ James T Jones TAH Kang GD Kerelska TH Kersey AD Khrebtukova I Kindwall AP Kingsbury Z Kokko-Gonzales PI Kumar A Laurent MA Lawley CT Lee SE Lee X Liao AK Loch JA Lok M Luo S Mammen RM Martin JW Mccauley PG Mcnitt P Mehta P Moon KW Mullens JW Newington T Ning Z Ng BL Novo SM O'neill MJ Osborne MA Osnowski A Ostadan O Paraschos LL Pickering L Pike AC Pike AC Pinkard DC Pliskin DP Podhasky J Quijano VJ Raczy C Rae VH Rawlings SR Rodriguez AC Roe PM Rogers J Bacigalupo MCR Romanov N Romieu A Roth RK Rourke NJ Ruediger ST Rusman E Sanches-Kuiper RM Schenker MR Seoane JM Shaw RJ Shiver MK Short SW Sizto NL Sluis JP Smith MA Sohna JES Spence EJ Stevens K Sutton N Szajkowski L Tregidgo CL Turcatti G Vandevondele S Verhovsky Y Virk SM Wakelin S Walcott GC Wang J Worsley GJ Yan J Yau L Zuerlein M Rogers J Mullikin JC Hurles ME Mccooke NJ West JS Oaks FL Lundberg PL Klenerman D Durbin R Smith AJ Accurate whole human genome sequencing using reversible terminator chemistry Nature 2008 456 7218 53 59 10.1038/nature07517 PMC2581791 18987734

[B5] Wang J Wang W Li R Li Y Tian G Goodman L Fan W Zhang J Li J Zhang J Guo Y Feng B Li H Lu Y Fang X Liang H Du Z Li D Zhao Y Hu Y Yang Z Zheng H Hellmann I Inouye M Pool J Yi X Zhao J Duan J Zhou Y Qin J Ma L Li G Yang Z Zhang G Yang B Yu C Liang F Li W Li S Li D Ni P Ruan J Li Q Zhu H Liu D Lu Z Li N Guo G Zhang J Ye J Fang L Hao Q Chen Q Liang Y Su Y San A Ping C Yang S Chen F Li L Zhou K Zheng H Ren Y Yang L Gao Y Yang G Li Z Feng X Kristiansen K Wong GKS Nielsen R Durbin R Bolund L Zhang X Li S Yang H Wang J The diploid genome sequence of an Asian individual Nature 2008 456 7218 60 5 10.1038/nature07484 PMC2716080 18987735

[B6] Kaiser J A Plan to Capture Human diversity in 1000 Genomes Science 2008 319 395 10.1126/science.319.5862.395 18218868

[B7] Service RF The Race for the $1000 Genome Science 2006 311 1544 1546 10.1126/science.311.5767.1544 16543431

[B8] Mardis ER ChIP-seq: welcome to the new frontier Nature Methods 2007 4 613 614 10.1038/nmeth0807-613 17664943

[B9] Hall N Advanced Sequencing Technologies and their Wider Impact in Microbiology The Journal of Experimental Biology 2007 209 1518 1525 10.1242/jeb.001370 17449817

[B10] Li H Ruan J Durbin R Mapping short DNA sequencing reads and calling variants using mapping quality scores Genome Res 2008 18 11 1851 8 10.1101/gr.078212.108 PMC2577856 18714091

[B11] Lin H Zhang Z Zhang MQ Ma B Li M ZOOM! Zillions of oligos mapped Bioinformatics 2008 24 21 2431 7 10.1093/bioinformatics/btn416 PMC2732274 18684737

[B12] Langmead B Trapnell C Pop M Salzberg S Ultrafast and memory-efficient alignment of short DNA sequences to the human genome Genome Biology 2009 10 3 R25 http://genomebiology.com/2009/10/3/R25 10.1186/gb-2009-10-3-r25 PMC2690996 19261174

[B13] Grumbach S Tahi F A new challenge for compression algorithms: Genetic sequences Information Processing & Management 1994 30 6 875 886

[B14] Matsumoto T Sadakane K Imai H Biological sequence compression algorithms Genome informatics 2000 11 43 52 11700586

[B15] Apostolico A Lonardi S Off-Line Compression by Greedy Textual Substitution Proceedings of the IEEE 2000 88 11 1733 1744

[B16] Chen X Li M Ma B Tromp J DNACompress: fast and effective DNA sequence compression Bioinformatics 2002 18 1696 1698 10.1093/bioinformatics/18.12.1696 12490460

[B17] Manzini G Rastero M A simple and fast DNA compressor Softw Pract Exper 2004 34 14 1397 1411

[B18] White WTJ Hendy MD Compressing DNA sequence databases with coil BMC Bioinformatics 2008 9 242 10.1186/1471-2105-9-242 PMC2426707 18489794

[B19] Christley S Lu Y Li C Xie X Human Genomes as Email Attachments Bioinformatics 2008 25 274 275 10.1093/bioinformatics/btn582 18996942

[B20] The gzip home page http://www.gzip.org

[B21] McEliece RJ The Theory of Information and Coding 1977 Reading, MA: Addison-Wesley Publishing Company

[B22] Cover TM Thomas JA Elements of Information Theory 1991 New York: John Wiley

[B23] Golomb SW Run-Length Encodings IEEE Transactions on Information Theory 1965 12 3 399 401

[B24] Elias P Universal Codeword Sets and Representations of the Integers IEEE Transactions on Information Theory 1975 21 2 194 203

[B25] Baldi P Benz RW Hirschberg D Swamidass S Lossless Compression of Chemical Fingerprints Using Integer Entropy Codes Improves Storage and Retrieval Journal of Chemical Information and Modeling 2007 47 6 2098 2109 10.1021/ci700200n PMC2536658 17967006

[B26] Huffman D A method for the construction of minimum redundancy codes Proc IRE 1952 40 1098 1101

[B27] Moffat A Stuiver L Binary Interpolative Coding for Effective Index Compression Inf Retr 2000 3 25 47

[B28] Moffat A Anh V Binary codes for locally homogeneous sequences Information Processing Letters 2006 99 175 180

[B29] Hirschberg DS Baldi P Effective Compression of Monotone and Quasi-Monotone Sequences of Integers Proceedings of the 2008 Data Compression Conference (DCC 08) 2008 Los Alamitos, CA: IEEE Computer Society Press in press

[B30] Rissanen JJ Langdonr GG Arithmetic coding IBM Journal of Research and Development 1979 23 2 149 162

[B31] Witten IH Neal RM Clearly JG Arithmetic Coding for Data Compression Communications of the ACM 1987 30 6 520 540

[B32] Kao MY Encyclopedia of Algorithms 2007 Secaucus, NJ, USA: Springer-Verlag New York, Inc

[B33] Witten I Moffat A Cell TB Managing Gigabytes: Compressing and Indexing Documents and Images 1999 Second Morgan Kauffman

[B34] Johnson DS Mortazavi A Myers RM Wold B Genome-wide mapping of in vivo protein-DNA interactions Science 2007 316 1497 1502 10.1126/science.1141319 17540862

[B35] Li G Ma L Song C Yang Z Wang X Huang H Li Y Li R Zhang X Yang H Wang J Wang J The YH database: the first Asian diploid genome database Nucleic Acids Res 2009 37 D1025 8 10.1093/nar/gkn966 PMC2686535 19073702

